# Online Health Communities: an alternative feasible data registry tool for developing countries

**DOI:** 10.1186/s12961-023-00976-w

**Published:** 2023-04-06

**Authors:** Omnia Abdelraheem, Diana G. Sami, Mohamed Salama

**Affiliations:** 1 Institute of Global Health, and Human Ecology (I-GHHE), The American University in Cairo, Cairo, Egypt; 2 Faculty of Medicine, Mansoura University, Mansoura, Egypt; 3 Global Brain Health Institute (GBHI), Trinity College Dublin, Dublin, Ireland

**Keywords:** Online Health communities: OHC, Data registry, Data collection, Disease cohorts

## Abstract

Given the many challenges facing healthcare access in many developing countries and the added limitations observed in emergencies like COVID-19 pandemic, the authors here discuss an alternative and feasible approach to overcome all these limitations.

A recent study by Adams and colleagues showed that medical teams should validate Online Health Communities (OHCs) as they do not threaten the parent–provider relationship [1]. The idea of OHCs is broadly accepted to increase engagement, disease awareness and management. Successful OHCs exist in many nations; in the USA, PatientsLikeMe has more than 850,000 members with more than 2800 diseases [2]. The UK has HealthUnlocked with 1.5 million members, covering more than 250 conditions [3]. More than 420 million people are registered on China's OHC Ping A Good Doctor, and the platform has close to 1.27 billion consultations overall [4].

The construction of health registries in developing countries has been challenging. Some developing countries suffer from a lack of disease registries and others face the failure of their registry projects. Significant barriers include; inconsistent documentation and archiving system (absence of Electronic Health Records), low quality of the data collected, lack of budget, scarcity of trained and qualified personnel, the poor performance of managers, low stakeholders' interest/motivation, and absence of funding [5–7].

Since internet users are significantly increasing in developing countries, for example, approximately 73% of the population in Egypt has access to the internet, the OHCs could provide an excellent opportunity to develop online disease registries [8]. They offer advantages to the healthcare sector in many ways, such as being accessible to the population, achieving high levels of engagement, and removing physical and location barriers [9]. These platforms offer patients customized disease-specific reports and visualization tools to help patients understand and share information about their condition which result in better disease management [10]. This improves the quality of life and decreases the sense of loneliness [11]. OHCs may offer a chance to replace conventional health information systems in the developing countries. They might serve as source of data for research, patient care, and policy shaping. Additionally, OHCs can help these countries overcome the issue of health illiteracy by supplying patients and caregivers with reliable information and knowledge [12]. In addition, they will offer new insights for researchers to learn about the processes and outcomes of care from a patient perspective [13]. As a result, patients will be shifted from passive recipients of medical care to experts in their conditions, leading to patient-driven innovations. Since social support is one of the fundamental motives behind individuals engaging in OHCs, the broad reach will attract governments and policymakers to consider disease registries and might end up as collaborators in these OHCs [14].

OHCs are considered a reliable and valid resource for medical research [15]. This is evidenced by hundreds of studies that have been published in peer-reviewed medical and scientific journals, which have validated the utility of OHCs as a research tool. PatientsLikeMe is one of the most OHCs with high publication output. The literature with OHCs data has enhanced our knowledge of various disease conditions. These include neurodegenerative diseases such as Amyotrophic Lateral Sclerosis (ALS) [16–18], Huntington’s disease [19], Parkinson’s disease [20–22], Multiple sclerosis [23–25], and Osteogenesis imperfecta [26], as well as neurological diseases such as Epilepsy [27, 28], Bipolar disorder, depression [29], and Insomnia [30]. Additionally, OHCs have also provided insights into autoimmune diseases like Rheumatoid Arthritis [31, 32], Neuromyelitis Optica [33], cancer types such as Non-Small Cell Lung Cancer [34], Ovarian Cancer [35], metabolic diseases such as Diabetes Mellitus [35, 36], and cardiovascular diseases [37]. Several studies revealed OHCs improved population health. One study showed that OHC played an important role in promoting healthy behavior. The results of the study showed that social integration support from online social relationships have a positive relationship with users’ health behavior and increased informational support [38]. Other studies indicated that OHC increased patient empowerment which consequently improved health outcomes [39]. Additionally, the studies also address various themes and perspectives such as patient perception [40], patients reported outcomes (PROs) [41], crowdsourcing, patient-centeredness [42, 43], pharmacovigilance and adverse drug reactions reporting [44], drug development process [45] and off-label prescribing [46].

OHCs also provide a unique opportunity for real-world data and observational studies. They are considered new tools for collecting and analyzing data for epidemiological research. Using Ping A Good Doctor, a cross-sectional survey study analyzed 35.3 million consultations and inquisitions over the course of 1 year [4]. The study found that the most frequently consulted departments were gynecology and obstetrics, dermatology, and pediatrics. The most common diseases were acute upper respiratory infections, pregnancy, and dermatitis. Most users were female and between the ages of 19 and 35. The study found that online healthcare services can relieve the stress on hospitals and provide good user experiences [47]. So, Online health communities can reduce the prevalence and incidence of diseases by providing access to accurate and up-to-date information about different health conditions and treatments. Also, OHCs are heavily used by patients with long- term conditions. It is thought that OHCs have potential to promote health, usage of healthcare resources, and facilitate self-management of illness [48]. Also, OHCs provided social support for ongoing health-related problems especially at the onset of COVID-19 pandemic [49].

Like any virtual community, privacy invasion or personal health information (PHI) disclosure is a crucial challenge facing the OHCs. Qualified researchers in universities from different disciplines can work collaboratively to manage the platform activities from data acquisition to data analysis, generate reports and publications, and maintain privacy. Also, it will open the door for pharmaceutical companies to understand patients' needs in a specific population leading to personalized medicine and better evaluating of drug effects [50].

Digital platforms have contributed to the spread of misinformation online, which can have a detrimental effect on people's health, as stated by the WHO [51]. Therefore, it is important to note that certain OHCs, such as PatientsLikeMe and HealthUnlocked, operate as patient-driven platforms where information is reported and shared by members [52, 53]. It is crucial to understand that the information provided within these communities does not replace the need for guidance and consultation from healthcare professionals. In adherence to this, terms and conditions of use for these types of communities typically stipulate that the reliance on any information provided is the responsibility of the individual member [54, 55]. It is also important to be aware of the limitations in distinguishing between credible and unreliable information from these patient-driven platforms, and to exercise caution when interpreting the information provided. In contrast, other online health communities such as Ping An Health, which is also known as Ping An Good Doctor, function as a national internet-based hospital and provide online healthcare services, which may have more rigorous standards for the information provided [47].

To combat misinformation, OHC should have a team of researchers and experts in the field to make sure that all information posted are from a reliable source with scientific evidence and platforms with evidence-based data [51]. Although artificial intelligence is used unethically to spread misinformation, it also plays a crucial role in fighting misinformation and infodemics [56]. With support from the government and acceptance by the public, online health care services could develop quickly and greatly benefit people's daily lives and help in solving health problems [47].

Although OHC is crucial for developing countries due to the economic situation, there are some limitations. Low-Middle Income Countries (LMICs) have limited access to the internet; about 35% of the population have access to the internet compared to 80% in the developed countries. According to the World Bank, "Connecting for inclusion, high-speed internet access is not a luxury, but a basic necessity for economic and human development in both developed and developing countries" [57].

In conclusion, OHCs offer an alternative and workable strategy to get over the restrictions of developing disease registries. Also, it provides an accessible solution for healthcare access in light of the numerous obstacles that many developing countries face in their health systems and during emergencies.

## Data Availability

Not applicable.
